# Impact of Different Drying Processes on the Physico-Chemical Properties of Liquitablet Formulations Containing Lornoxicam

**DOI:** 10.3390/pharmaceutics17091096

**Published:** 2025-08-22

**Authors:** Csilla Balla-Bartos, Alaa Gamiel, Anett Motzwickler-Németh, Rita Ambrus

**Affiliations:** Faculty of Pharmacy, Institute of Pharmaceutical Technology and Regulatory Affairs, University of Szeged, Eötvös Utca 6, 6720 Szeged, Hungary; bartos.csilla@szte.hu (C.B.-B.); gamiel.alaa@stud.u-szeged.hu (A.G.); nemeth.anett@szte.hu (A.M.-N.)

**Keywords:** lornoxicam, in vitro dissolution, liquitablets, freeze-drying, vacuum-drying

## Abstract

**Background**: Enhancing bioavailability is the target of most pharmaceutical research; this can be achieved by modifying the physico-chemical characteristics of poorly water-soluble drugs intended for oral administration using different techniques. The preparation of liquitablets by blister molding technique provides an opportunity to increase the bioavailability of the drug using an optimal combination of release-facilitating additives. Lornoxicam is an effective non-steroidal anti-inflammatory drug with low water solubility. This study aimed to formulate a novel lornoxicam-containing liquitablets. The effect of different drying techniques on the physico-chemical properties and in vitro dissolution of lornoxicam was investigated. The physical parameters of the tablets were also studied. **Methods**: The additives applied in the formulation included Tween^®^ 80, Polyvinylpyrrolidone (PVP K90), Avicel^®^ PH-102, and sodium bicarbonate. Vacuum-drying and freeze-drying were employed to produce liquitablets. The influence of various drying methods on crystallinity and intra- and interparticle phenomena was investigated. In Vitro dissolution tests were performed at pH 1.2, and a comparison was made between our products and commercial tablets using the pairwise similarity factor model (f2). **Results**: The liquitablets demonstrated high hydrophilicity and a lower crystallinity of the drug. Freeze-dried liquitablet showed improved dissolution compared to that of the pure drug or the vacuum-dried product. A similarity was observed between our freeze-dried product and the marketed fast-release tablets. **Conclusions**: This research demonstrates that preparation of liquitablet in combination with freeze-drying has a significantly positive effect in improving the in vitro dissolution rate of lornoxicam.

## 1. Introduction

In recent years, there has been growing interest in developing new pharmaceutically active ingredients. It is worth mentioning that about 40% of approved drugs and nearly 90% of the newly developed chemical entities under development are poorly water-soluble compounds [1,2]. These insoluble drugs are associated with many problems, such as slow dissolution rate, suboptimal plasma concentration, and poor drug bioavailability [3], which challenge drug development in addition to difficult and complex formulation processes [4]. Several physical and chemical approaches have been used by scientists in pharmaceutical technology to overcome these problems and produce effective drug formulations, such as formation of prodrug [5,6], solubilization by cosolvency [7], co-crystal and salt formation [8,9], particle size reduction (micronization and nanonization) [10,11,12], complexation [13,14], lipid-based formulation [15] and amorphization using solid dispersion [16,17,18]. Preparation of liquitablets, a recently developed dosage form [19], is also a possibility to improve the bioavailability of the drug, which requires the use of appropriate solid and liquid liberation modulators. The increased dissolution rate of the drug can be attributed to the hydrophilic character of additives used for liquitablet preparation. In such a system, liquid vehicles can be polyethylene glycols, Propylene glycol, and Tween^®^ 85 [20], the solid carrier could be microcrystalline cellulose [19,20], and sodium bicarbonate as a microenvironmental pH-modifier [19]. Moreover, the employment of hydrophilic polymers such as polyvinylpyrrolidone (PVP), recognized for its binder functionality to achieve a balance between the tablet’s mechanical strength and an acceptable level of friability [21]. Formulation of liquitablets can also be implemented by direct compression and blister molding technique. Blister molding process is a simple and cost-effective method for drug formulation. There is literature on the preparation of liquitablets containing different drugs, such as non-steroidal anti-inflammatory drugs (NSAIDs): naproxen [19] and ketoprofen [20], hydrochlorothiazide [22], etc.

Lornoxicam (LXM) is a potent oxicam NSAID with analgesic, anti-inflammatory, and antipyretic pharmacological actions [23]. It shows remarkable efficacy in controlling presurgical and post-surgical pain associated with gynecological, orthopedic, abdominal, and dental procedures. It is available on the market as oral and parenteral formulations in doses of 4–8 mg and 4 mg/mL, respectively. However, its utility is restricted by its poor solubility and short half-life (3–5 h) [24]. Several strategies have been studied to improve the dissolution rate and oral bioavailability, thereby reducing side effects of LXM, such as solid dispersion [25], self-emulsifying system [26], complexation by inclusion in β-cyclodextrin [27], co-crystallization [28], and the use of a nanocarrier [29,30]. The conventional liquisolid technique has been applied to LXM, using propylene glycol or polyethylene glycol 400 as a solvent, and microcrystalline cellulose and silicon dioxide were used as additives, primarily to achieve sustained drug release [31]. LXM is widely used in Europe and other global regions; however, its formulation choices are comparatively constrained when juxtaposed with other NSAIDs. The development of cost-effective, stable, and patient-centric formulations has the potential to improve accessibility and therapeutic outcomes in various markets. In our previous work, we prepared blister-packed immediate release liquitablets with enhanced dissolution rate and reduced gastric irritation in vivo. The investigation of the combination of excipients (wetting agent, binder, solubilizer, pH-modifier, carrier) was chosen to obtain the optimum composition [32]. To prepare liquitablets, drying was applied at room temperature, which required a prolonged period (7–10 days).

In this study, our objective is to examine–as a preformulation study–the impact of various drying methods, specifically vacuum-drying and freeze-drying, on the physico-chemical properties and in vitro dissolution of LXM in an oral liquitablet, which was prepared without the use of organic solvents. Consequently, this preformulation study contributes to pharmaceutical research on both LXM and the blister molding technique by presenting an oral formulation of improved solubility with minimum gastric side effects.

## 2. Materials and Methods

### 2.1. Materials

The active pharmaceutical ingredient (API) was LXM (6-Chloro-4-hydroxy-2-methyl-N-(pyridin-2-yl)-2H-thieno [2,3-e][1,2]thiazine-3-carboxamide 1,1-dioxide) (Tokyo Chemical Industry, Tokyo, Japan). Polyvinylpyrrolidone K90 (PVP K90) (Sigma-Aldrich, Shanghai, China) used as a binder, Tween^®^ 80 (Sigma-Aldrich Chemie GmbH, Darmstadt, Germany) as a wetting agent and liquid solvent, sodium bicarbonate (Sigma-Aldrich Co., Ltd., Budapest, Hungary), used as an alkanizer, and Avicel^®^ PH-102 (FMC Corporation, Philadelphia, PA, USA) as a carrier and solid matrix producer were chosen as excipients and purified water was used to prepare the aqueous solutions.

### 2.2. Methods

#### 2.2.1. Solubility Studies as Preliminary Experiments

For the solubility test, different solvents and the effect of solubility regulators—chosen based on our previous experiences—were investigated. An excess amount of LXM was introduced into screw-capped bottles containing each solvent (distilled water at pH 6.8, buffer solution at pH 1.2, pure Tween^®^ 80, and a mixture of Tween^®^ 80 and a 15% *w*/*v* aqueous solution of NaHCO_3_ in a 3:1 volume ratio). The mixtures were subjected to magnetic stirring at a temperature of 25 ± 5 °C for a duration of 24 h. Subsequently, the samples were filtered, and the filtrate was analyzed using spectrophotometric methods (UV/vis spectrophotometry with an UV/VIS Spectrophotometer (ATI-Unicam, Cambridge, UK) at 375 nm to determine the drug concentration [32].

#### 2.2.2. Sample Preparation

**Figure 1:**
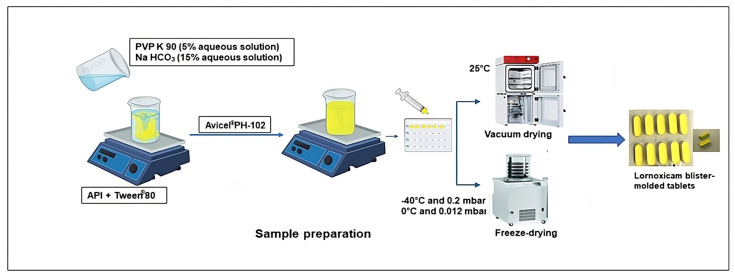
Preparation of freeze-dried and vacuum-dried samples.

For the preparation of LXM liquitablets, first, the drug was dissolved in the optimized mixture of solid and liquid release modulators (Figure 1): Tween^®^ 80 is utilized due to its substantial ability to enhance the solubilization of drugs with poor solubility [33] and its low cytotoxicity [34]; PVP K90, although predominantly used as a binder [35], demonstrates multifunctional characteristics, including inhibition of drug recrystallization during the dissolution process [36]; and Avicel^®^ PH-102, in addition to its role as a carrier [20,35], is recognized for its wicking capacity, which contributes to the enhancement of dissolution [37]. The composition of the prepared products is demonstrated in Table 1. To produce a sample set consisting of 10 solid tablets per 10 mL solution, the dispersion of 80 mg LXM in 2 mL of Tween^®^ 80 was prepared using a magnetic stirrer. An aqueous solution containing PVP K90 (5% *w*/*v*), which was found to yield the most effective results in maintaining tablet integrity in our previous study, and sodium bicarbonate (15% *w*/*v*), which was determined to provide optimal solubility [32] were prepared and subsequently incorporated into the drug and Tween^®^ 80 mixture, as a wetting agent, followed by the addition of Avicel^®^ PH-102 [38]. The resultant liquid mixtures were filled into blisters and subjected to a drying process, utilizing either vacuum-drying in a Binder VDL drying oven (Binder GmbH, Tuttlingen, Germany at 25 °C for 24 h or a freeze-dryer. Blister was then placed into a laboratory freeze-drying apparatus, Scanvac CoolSafe 100–9 Pro (LaboGeneApS, Lynge, Denmark), equipped with a Rotary Vane vacuum pump (Vacuubrand RZ 2.5, Wertheim, Germany). The apparatus was pre-frozen to maintain the sample trays within the drying chamber at −40 °C. Process parameters were continuously monitored and recorded via the software (Scanlaf CTS16a02, version 7) at a cycle setting of −40 °C and 0.2–0.5 mbar for 36 h within a vacuum oven dryer. The primary drying phase was conducted at −40 °C and 0.012 mbar for 16 h, followed by a secondary drying phase at 0 °C and 0.012 mbar for 2 h based on the method used by our research laboratory group. After 36 h, liquitablets were obtained.

The reference physical mixture (PM) was formulated by combining LXM with the additives (Tween^®^ 80, Polyvinylpyrrolidone (PVP K90), Avicel^®^ PH-102, and sodium bicarbonate) in proportions identical to those utilized in the formulation, at 50 rpm for 10 min employing a shaker mixer (Turbula System Schatz; Willy A. Bachofen AG Maschinenfabrik, Basel, Switzerland). It should be noted that the liquid constituent, Tween^®^ 80, was completely adsorbed by the solid carrier, Avicel^®^ PH-102, facilitating uniform assimilation and distribution throughout the mixture.

#### 2.2.3. Liquitablets Characterization

##### Liquitablets Parameters

Three tablets of each formula were individually weighed, and the average weight and the mass difference were calculated. The width and length of the tablets were determined using a screw micrometer (Mitutoyo Corp., Tokyo, Japan).

##### Differential Scanning Calorimetry (DSC) Thermogravimetric Analysis (TGA)

The thermal properties of the products (VDLT and FDLT) were examined and compared with those of API and PM through the interpretation of the DSC and TGA patterns, utilizing the Mettler Toledo DSC 821e thermal analysis system equipped with the STARe SW 16.3 thermal analysis software version 9.0 provided by Mettler Toledo (Mettler Inc. Schwerzenbach, Switzerland). Approximately 2–5 mg of samples were hermetically sealed in flat-bottom aluminum pans and heated at a constant rate of 10 °C/min in an atmosphere of nitrogen in a temperature range of 20–400 °C [39].

##### X-Ray Powder Diffraction Analysis (XRPD)

API, physical mixture of API-excipient (PM), vacuum-dried product (VDLT), and freeze-dried product (FDLT) were evaluated by powder X-ray diffraction analysis (XRPD) (BRUKER D8 Advance Diffractometer, Bruker AXS GmbH, Karlsruhe, Germany) to evaluate solid-state physical structure. Approximately 10 mg of the sample material was finely ground prior to deposition in the holder. The experimental parameters were established as follows: Cu Kλ1 radiation (λ = 1.5406 Å) constituted the radiation source, and the samples were scanned at temperatures of 40 kV and 40 mA in an angular range of 3–40° 2-Theta (2θ). A temporal resolution of 0.1 s per step was employed with an increase in step of 0.01°, and the calibration of the X-ray instrument was performed using standard aluminum oxide powder. The crystallinity index (*Xc*%) for each sample was quantitatively ascertained utilizing Equation (1) via the DIFFRAC.EVA.V5.2 software and data from the Cambridge Structural Database were used as a reference.

(1) $$Xc\%=\frac{A_{crytalline}}{A_{total}}\times 100\;\;\;$$

where

*Xc*% is the crystallinity index,

*A_crystalline_* refers to the area under the crystalline peaks, and

*A_total_* indicates the total area of the diffractogram.

##### Fourier Transform Infrared Spectroscopy (FT-IR)

The spectra of the products (VD and FDLT) were obtained and compared to the observed peaks of the API and PM spectra using Fourier transform infrared spectroscopy (AVATAR 330 FT-IR; Thermo Nicolet, Unicam Hungary Ltd., Budapest, Hungary). The samples were ground with 0.15 g of potassium bromide and compressed into a disc with 10-ton pressure. The scan was performed in the range of 4000 to 400 cm^−1^ and with a resolution of 4 cm^−1^. The rsecorded bands are identified for the functional group.

##### Wettability

The surface free energy and polarity of the Active Pharmaceutical Ingredient (API) and the products (VD and FDLT) were calculated. Approximately 0.10 g of the samples were subjected to compression using a 1-ton hydraulic press (Perkin Elmer hydraulic press, Specac Inc., Waltham, MA, USA). Six pastilles were obtained from each sample. Subsequently, the surface of the pastilles was treated with polar solvents (4.8 µL of purified water) and non-polar solvents (2.0 µL of diiodomethane). The contact angle was measured using the Dataphysics OCA 20 apparatus (Dataphysics Inc. GmbH, Filderstadt, Germany) over an interval of 1 to 25 s. The contact angles for the two fluids were obtained successfully. The surface free energy (*γs*) of the products, comprising the polar component (*γsp*) and the dispersive component (*γsd*); (*γs* = *γsp* + *γsd*), was determined based on the application of the Wu equation. The surface tension values for the employed liquids can be referenced in the literature: distilled water *γp* = 50.2 mN/m, *γd* = 22.6 mN/m and diiodomethane *γp* = 1.8 mN/m, *γd* = 49 mN/m. The Wu equation (Equation (2) is illustrated below, where θ denotes the contact angle, *γ* represents the surface free energy, *s* signifies the solid phase, *l* denotes the liquid phase, d denotes the dispersion component, and *p* represents the polar component.

(2) $$\left(1+\cos\theta \right)\gamma_{l}\;=\frac{4({\gamma_{s}}^{d}{\gamma_{l}}^{d})}{{\gamma_{s}}^{d+}{\gamma_{l}}^{d}}+\frac{4({\gamma_{s}}^{p}{\gamma_{l}}^{p})}{{\gamma_{s}}^{p+}{\gamma_{l}}^{p}}$$

The polarity (Pol) was calculated as the ratio between the surface free energy of the polar component and the surface free energy multiplied by 100 (Equation (3)).

(3) $$\mathrm{Pol}=\frac{\gamma^{p}}{\gamma^{s}}\times 100$$

#### 2.2.4. In Vitro Dissolution Test

The dissolution test was carried out using the USP dissolution apparatus I (instrument) at 50 rpm. Weight equivalent to the dose (8 mg) was placed in the dissolution vessel containing 100 mL of simulated gastric medium (pH 1.2), maintained at 37 ± 0.5 °C. Aliquots from the dissolution medium were withdrawn at specific time points (5, 10, 15, 30, and 60 min), and the withdrawn samples were replaced with equal volumes of fresh medium to keep the dissolution volume constant throughout the experiment. The samples were filtered through a 0.45 µm Millipore filter (Millex-HV filter unit, Millipore Corporation, Bedford, MS, USA) and analyzed using a UV spectrophotometer at the predetermined λmax (374 nm) using a buffer of pH 1.2 as a blank. Each test was performed in triplicate, and average values and standard deviations were recorded. The drug concentration values were corrected for progressive dilution to obtain the cumulative amount dissolved using Equations (4) and (5) derived from the Wagner model for dissolution rate calculation [40]:

(4) $$Q^{\prime }t\left(n\right)=Vr\times Cn+Vs\times \Sigma \;Cm$$

(5) $$Cumulative\;\%\;released=\frac{\left(Q^{\prime }t\left(n\right)\right)}{dose}\times 100$$

where *Q′t (n)* is the current cumulative mass of the drug dissolved at time t.

*Cn* represents the current concentration in the dissolution medium.

*Σ Cm* denotes the total sum of the previous measured concentration.

*Vr* is the volume of the dissolution medium and

*Vs* corresponds to the volume of the sample removed for analysis.

The dissolution data obtained were plotted as the cumulative percentage of the drug dissolved as a function of time. The dissolution profiles of the API, PM and products (VD and FDLT) were compared through the calculation of the model-independent similarity factor (*f_2_*), which can be defined by Equation (6) [41]:

(6) f2=50×log1+1n∑j=1nRj −Tj2−0.5×100

Furthermore, the freeze-dried liquitablets were compared to the marketed product (simple formulation (SM) and rapid formulation (RM)).

#### 2.2.5. Statistical Analysis

Statistical analyses were conducted using the GraphPad Prism 5.0.1 software (GraphPad Software, San Diego, CA, USA). A two-tailed *t*-test with measurements carried out in triplicate was performed for comparing crystallinity, and analysis of variance (ANOVA) for dissolution rate tests, in which the *p*-values < 0.05 denoted statistically significant differences.

## 3. Results and Discussion

### 3.1. Solubility Studies

The saturated solubility of LXM in distilled water, pH 1.2, and in the presence of different excipients is shown in Table 2. It is evident that LXM has poor solubility in pH 1.2 (0.003 mg/mL); this is due to the acidic nature of LXM, which renders it unionized at low pH. In addition, the crystalline structure could reduce the affinity of hydrogen bond formation with water, making it insoluble. Furthermore, LXM exhibits slight lipophilicity, demonstrated by an apparent partition coefficient of 1.8 in an n-octanol/buffer system at pH 7.4 and having a pKa of 4.7. Due to keto-enol tautomerism, where the presence of a basic pyrimido group with a pKa of 5.5 enhances the acidity of the enolic group to a pKa between 1 and 2, LXM becomes amphoteric, existing as a zwitterion within the physiological pH range of 2–5, and transitions to an anionic form at pH values of 6 and higher [42]. The application of Tween^®^ 80—pH 6. 7—also improves solubility by forming micelles and facilitating the dissolution process; therefore, the solubility of LXM in Tween^®^ 80 increased to 6.197 mg/mL. The application of an aqueous solution of alkalizing sodium bicarbonate (15% *w*/*v*) demonstrated solubility enhancement as well (5.794 mg/mL). Consequently, both Tween^®^ 80 and sodium bicarbonate were incorporated into the formulation. The combination of these two solubility enhancer additives resulted in the highest solubility value of LXM: 16.56 mg/mL.

### 3.2. Liquitablets Parameters

All liquitablets showed physical integrity, showing no signs of damage or fragmentation during handling. Each liquitablet was successfully and completely removed from the blister packaging without adhesion. The mean percentage of LXM content, liquitablet dimensions, and average weights are shown in Table 3. All liquitablets adhered to the pharmacopeial requirements of the average weight (weight variation range of ±5%), which was approximately 0.306 g in both cases [43]. The drug content was above 95% for both products. The liquitablets were about 23 mm long and 9 mm wide.

### 3.3. Solid Phase Characterization

#### 3.3.1. Thermoanalytical Results

DSC was employed to investigate the thermal behaviors of LXM in the raw form, in the physical mixture, and in the products prepared with different drying processes.

**Figure 2:**
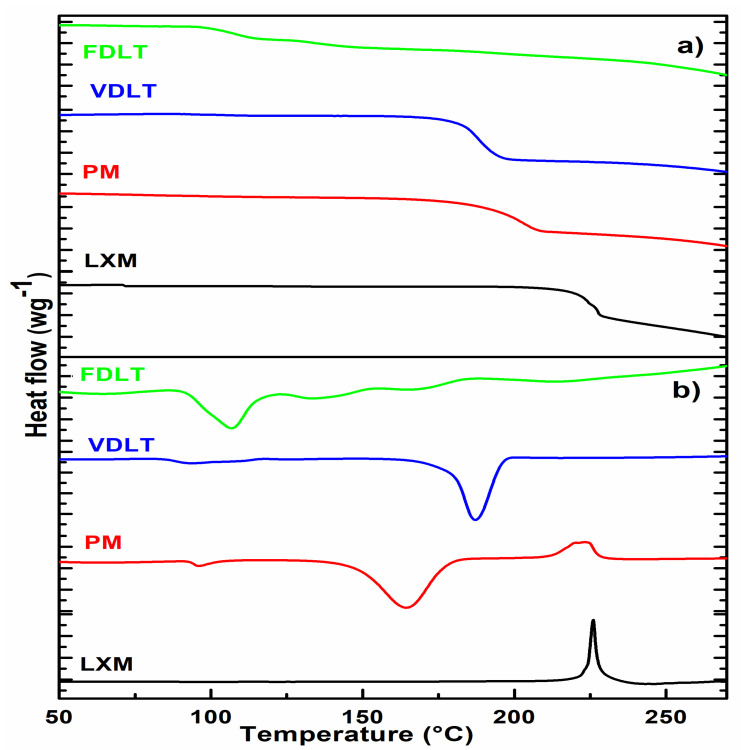
(**a**) DSC and (**b**) TGA curves of API, physical mixture, vacuum-dried, and freeze-dried liquitablets.

Combining DSC and TGA will provide a prediction of the effect of processing on drug stability [44]. DSC curve of LXM did not exhibit any endothermic melting peak; nevertheless, thermal decomposition transpired concurrently, as indicated by a sharp exothermic peak occurring at 225.48 °C, which is further corroborated by the TGA. Our thermal findings of LXM aligned entirely with previously published reports [45,46,47]. In the case of a physical mixture and vacuum-dried product, the thermal decomposition of LXM occurred at a lower temperature compared to that of the pure drug, which is also supported by the weight loss on the TGA curve at the same temperature. In the prepared products, DSC analysis identified a glass transition temperature (Tg) with a midpoint (ISO) of 112.35 °C, and a range extending from 108.26 °C to 116.41 °C. The limited transition range (ΔTg = 8.15 °C) implies the presence of an amorphous phase. TGA decomposition of the freeze-dried tablet is slowly indicating more stability than the vacuum-dried powder, which can be explained by the low moisture content (usually ≤ 1%) of the freeze-dried product (Figure 2) [48].

#### 3.3.2. Structural Characterization: X-Ray Powder Diffraction Analysis (XRPD)

**Figure 3:**
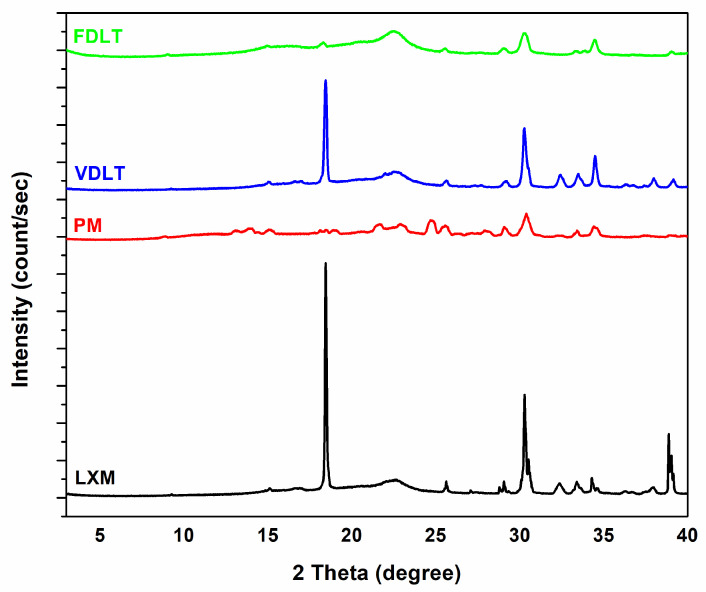
XRPD diffractograms of API, physical mixture, vacuum-dried powder, and freeze-dried powder.

The crystallinity of API and products after processing was assessed using XRPD patterns (Figure 3). The XRPD pattern of LXM clearly exhibits sharp multiple diffraction lines at (2θ) angles of 8.77°, 13.48°, 14.33°, 15.08°, 18.91°, 20.52°, 21.53°, 22.96°, 24.68°, 25.43°, 28.05°, 30.50°, and 44.64°, which are indicative of the crystalline characteristics of LXM [49]. Notwithstanding the crystalline predominance of the API utilized in this study, it is plausible that a minor fraction of amorphous material is present. This could be attributed to the intrinsic properties of the material, conditions of synthesis, or subtle processing influences preceding the analytical evaluation, such as milling or storage. Conversely, a reduction in the intensities of diffraction lines within the sample diffractograms suggests a diminishment in the crystallinity of the drugs [50], and a computed *p*-value = 0.0001 demonstrates statistical significance at the 95% confidence level. Table 4 shows the crystallinity percentage. The crystallinity of LXM in the physical mixture decreased compared with the raw drug, which can be explained by the presence of the PVP K90, which is an amorphous polymer [51]. In the case of the freeze-dried product, only approximately 50% of the drug was crystalline. This phenomenon can be explained by the conditions of freeze-drying (fast freezing, vacuum sublimation), which prevent the formation of crystalline structure, and also by the PVP, which can help prevent the drug from crystallizing, maintaining it in a more soluble amorphous state [52]. Crystalline drugs are generally characterized by reduced solubility attributable to the robust intermolecular forces that must be surmounted during dissolution, as evidenced by their highly packed lattice configurations. Conversely, amorphous drugs demonstrate superior solubility due to their elevated free energy and a disordered molecular arrangement, thereby accelerating dissolution rates [53,54,55].

#### 3.3.3. Structural Characterization: FT-IR

**Figure 4:**
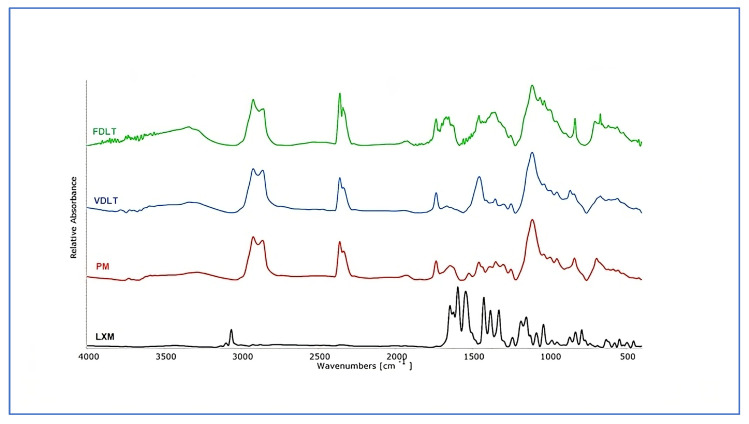
FT-IR spectra of API, physical mixture, freeze-dried, and vacuum-dried samples.

FT-IR spectroscopy was employed to investigate potential interactions between the LXM and the excipients used in the preformulations. The pure LXM FT-IR spectrum (Figure 4) showed weak and sharp absorption bands in the 3005.6–3133.0 cm^−1^ region, corresponding to aromatic H stretch vibrations. Additional bands were observed at 1645.8 and 1594.3 with 1503.3 cm^−1^, attributed to the stretching vibration of the carbonyl group in the primary amide and to the bending of the N–H in the secondary amide, respectively. Stretching vibrations of the sulfonyl group were detected at 1185.4 and 1382.4 cm^−1^ bands with weak intensities in the 690–870 cm^−1^ range were mostly related to aromatic C–H vibrations, of which the band at 789.7 cm^−1^ indicated the presence of C–Cl stretching [56,57]. These characteristic bands were partially present in the PM, especially within the fingerprint region, which confirmed the presence of the LXM. The spectrum of PM appeared as a superposition of the raw materials with no shifts in band positions, suggesting the absence of any chemical interaction between LXM and the excipients. Furthermore, the FT-IR spectra of the dried preformulations were identical to those of the PM, indicating that no new intermolecular interactions occurred during the VDLT or FD processes. Additionally, no spectral changes were observed that would suggest decomposition or structural changes at the molecular level in any of the samples.

#### 3.3.4. Wettability Investigations

Contact angle analysis was performed to determine the polarity of the materials. Table 5 presents the calculated polarity and surface free energy derived from contact angle measurements of LXM and the products. The correlation among contact angle, surface free energy, and hydrophilicity has been documented in several studies [58,59]. Our results showed that the dried products had decreased contact angle values with water (θ water) (for VDLT: 28.76°, and for FDLT: 33.60°), indicating a more hydrophilic character compared to the hydrophobic raw drug (42.0°). Additives (PVP K90, Tween^®^ 80, sodium bicarbonate, and Avicel^®^ PH-102), applied for the formulation of blister-molded liquitablets, were hydrophilic materials, increasing the wettability. The polarity of the products, calculated as the ratio of surface free energy of the polar component and the surface free energy, also increased, implying enhanced dissolution outcomes.

### 3.4. Evaluation of In Vitro Dissolution Test

**Figure 5:**
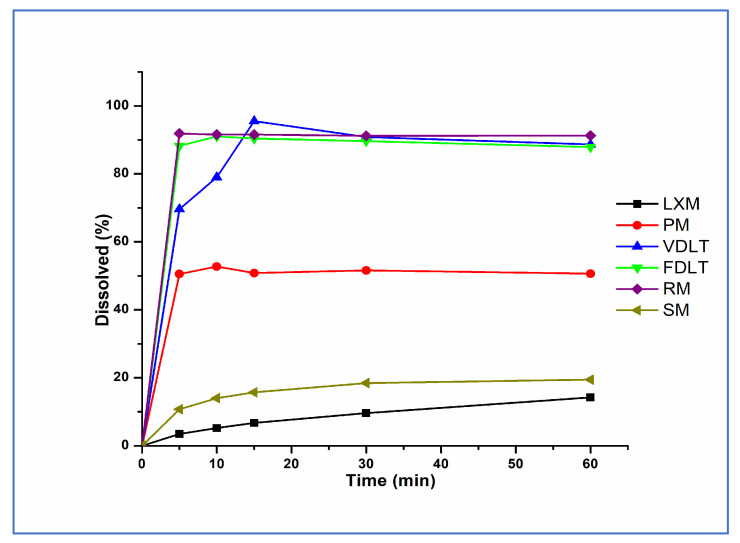
Dissolution rate of LXM, physical mixture, freeze-dried, vacuum-dried samples, and marketed formulations in pH 1.2.

Dissolution profiles of API, prepared formulations, and marketed products in the simulated gastric medium (pH 1.2) are illustrated in Figure 5. Our liquitablets showed complete dissolution of LXM in less than 15 min compared to <10% dissolution of pure drug. This improvement in dissolution rate and dissolved amount could be attributed to the effect of Tween^®^ 80, PVP K90, and the creation of a basic micro-environment with the sodium bicarbonate solution. Tween^®^ 80 and PVP, as non-ionic stabilizers with amphiphilic groups, provided steric stabilization dominated by the wetting effect [60]. Furthermore, PVP kept LXM in an amorphous form. The alkaline medium, provided by sodium bicarbonate, promoted the dissolution of the acidic drug as a result of partial ionization of LXM [61]. Additionally, there is a significant positive correlation between this observation and the formulation technique we used. Both drying techniques have positive effect on dissolution; however, freeze-drying has better results regarding time as the difference (Δ) is seen at 5 min (Δ = 18.62%) and 10 min (Δ = 12.01%), this fits well with Taldaev et al. in their review [62] and could be attributed to the amorphous structure of the LXM.

Our freeze-dried liquitablets were compared with simple (SM)- and rapid-marketed (RM) tablets containing LXM, as well. The dissolution of the SM product was slow, and only about 20% of LXM was liberated in the gastric fluid in the first 60 min. The dissolution of the freeze-dried product was like the RM tablet; over 80% of the active ingredient was released in the first 5 min and 100% within 10 min in both cases. This result was also supported by the similarity factor (*f_2_*), calculated by the statistical pairwise model (Table 6). The analysis of variance revealed a *p*-value of 0.0082, indicating a statistically significant difference. In order to consider similar dissolution profiles, *f_2_* values should be higher than 50 (50–100) [63]. Since *f_2_* was found to be 79.35 for the FDLT in comparison with the RM tablet, it can be stated that the release was similar to the RM formulation.

Several studies have linked decreased crystallinity and increased wettability to enhanced dissolution. For example, Jadhav and Yadav demonstrated that the enhancement of the drug’s surface wettability through solid dispersion and reducing drug crystallinity leads to a concomitant increase in solubility and bioavailability [55]. These findings are consistent with those reported by Rajebahadur et al., who demonstrated that the enhancement of solubility may arise from a partial transformation of the crystalline drug into the amorphous state, increase in the wettability of the drug by water-soluble polymers, improved separation of drug particles, micellar solubilization of the drug facilitated by elevated concentrations of surfactant polymers, and interaction between polymer and drug at the molecular level [64].

The current study is limited by the lack of in vivo investigations designed to assess gastric irritation reduction in animal models, which was examined in a preceding study that serves as the basis for this work. Furthermore, the absence of a long-term stability study of this formulation constrains the conclusions about shelf-life. Future research efforts will focus on performing in vivo studies to verify the reduction in gastric irritation, along with stability testing to ascertain the formulation’s appropriateness for clinical application.

## 4. Conclusions

In our work, liquitablets were prepared by applying two different drying methods. The effects of vacuum-drying and freeze-drying processes on the characteristics of blister-molded tablets were investigated. The size and weight of the liquitablets were examined. As a model drug, LXM, a poorly water-soluble NSAID, was used, and its solubility test was first carried out in different media. The effect of drying techniques on the physico-chemical properties of the products was determined. The results of the dissolution tests were compared and evaluated. Our study provides further evidence that the solubility of LXM could be successfully improved in acidic media by applying the appropriate additives. An important implication is that freeze-drying resulted in a product with high wettability (θ water = 33.60°) and the decreased crystallinity of the drug (≈50%). No chemical interaction between LXM and the excipients, and no new intermolecular interactions were detected during the drying procedures. Preparation of liquitablets with both drying methods had a prominent effect on the dissolution rate (100% in the first 15 min) compared with the pure drug and physical mixture. Freeze-dried product showed a similar dissolution curve as the rapid-marketed product (*f_2_* = 66).

Together, the formulation of liquitablets using the blister molding technique may provide a solution to the challenges of poorly water-soluble active ingredients. Because water is used as a dispersion medium, the procedure is considered a green technology, while the product is organic solvent-free. The findings of our research suggest the possibility of a supergeneric formulation as an advanced oral dosage form of LXM with rapid dissolution. A combination of solid and liquid additives makes the usage of much fewer excipients possible than those found in the marketed products.

## Figures and Tables

**Table 1:** Composition of prepared physical mixture (PM), freeze-dried (FDLT), and vacuum-dried (VDLT) samples.

	Composition/Tablet (% *w*/*w*)
Avicel^®^ PH-102	47.7
PVP K 90	5
Sodium bicarbonate	15
Tween^®^ 80	32.3

LXM content is 8 mg in each tablet.

**Table 2:** Solubility of LXM in different media.

Media	mg/mL (*n* = 3)
Distilled water (pH 6.8)	0.0131 ± 0.52
Buffer solution (pH 1.2)	0.003 ± 0
Pure Tween^®^ 80	6.197 ± 0.22
15% *w*/*v* aqueous solution of NaHCO_3_	5.794 ± 0.59
Tween^®^ 80: 15% *w*/*v* aqueous solution of NaHCO_3_ (3:1)	16.56 ± 0.94

**Table 3:** Parameters of freeze-dried and vacuum-dried tablets.

	Average Weight (mg) (*n* = 3)	Drug Content (%) (*n* = 3)	Dimensions (mm)	Image
VDLT	306 ± 0.94	95.73 ± 2.676	22.802 × 8.724	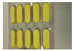
FDLT	307 ± 0.42	99.45 ± 3.950	22.819 × 8.724	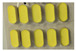

**Table 4:** Crystallinity percent calculated for API, PM, vacuum-dried, and freeze-dried samples.

	Crystallinity (%)
LXM	88.1
PM	66.50
VDLT	57.6
FDLT	50.3

**Table 5:** The polarity, the surface free energy (γ), and the contact angle values of API, freeze-dried, and vacuum-dried samples.

	Contact Angle (°)		Surface Free Energy (mN/m)	Polarity (%)
	Water	DIM		
API	42.0	18.68	65.37	33.32
VDLT	28.76	14.14	75.96	40.95
FDLT	33.60	16.01	73.19	39.75

**Table 6:** The similarity factor calculated for the dissolution rate of freeze-dried and vacuum-dried, freeze-dried, and simple and rapid-marketed products.

	Similarity Factor (*f_2_*)
FDLT and VDLT	49
FDLT and SM	7.45
FDLT and RM	79.35

## Data Availability

The original contributions presented in the study are included in the article; further inquiries can be directed to the corresponding author.

## Abbreviations

The following abbreviations are used in this manuscript:

API	active pharmaceutical ingredient
LXM	lornoxicam
PVP K90	polyvinylpyrrolidone K90
PM	physical mixture
VDLT	vacuum-dried liquitablets
FDLT	freeze-dried liquitablets
*w*/*w*	weight per weight
DSC	differential scanning calorimetry
TGA	thermogravimetric Analysis
Tg	glass transition
XRPD	X-ray powder diffraction analysis
FT-IR	Fourier transform infrared spectroscopy
min	minute
mm	millimeter
SM	simple marketed
RM	rapid marketed

## References

[1] Kalepu S., Nekkanti V. (2015). Insoluble drug delivery strategies: Review of recent advances and business prospects. Acta Pharm. Sin. B.

[2] Gowthami B., Krishna S.G., Rao D.S. (2020). Novel approaches to enhance oral bioavailability of poorly soluble drugs. Int. J. Res. Pharm. Sci..

[3] Nyamba I., Sombié C.B., Yabré M., Zimé-Diawara H., Yaméogo J., Ouédraogo S., Lechanteur A., Semdé R., Evrard B. (2024). Pharmaceutical approaches for enhancing solubility and oral bioavailability of poorly soluble drugs. Eur. J. Pharm. Biopharm..

[4] Kumari L., Choudhari Y., Patel P., Das Gupta G., Singh D., Rosenholm J.M., Bansal K.K., Das Kurmi B. (2023). Advancement in Solubilization Approaches: A Step towards Bioavailability Enhancement of Poorly Soluble Drugs. Life.

[5] Karbasi A.B., Barfuss J.D., Morgan T.C., Collins D., Costenbader D.A., Dennis D.G., Hinman A., Ko K., Messina C., Nguyen K.C. (2024). Sol-moiety: Discovery of a water-soluble prodrug technology for enhanced oral bioavailability of insoluble therapeutics. Nat. Commun..

[6] Stella V.J., Nti-Addae K.W. (2007). Prodrug strategies to overcome poor water solubility. Adv. Drug Deliv. Rev..

[7] Millard J.W., Alvarez-Núñez F., Yalkowsky S.H. (2002). Solubilization by Cosolvents: Establishing Useful Constants for the Log-Linear Model. Int. J. Pharm..

[8] Chettri A., Subba A., Singh G.P., Bag P.P. (2024). Pharmaceutical co-crystals: A green way to enhance drug stability and solubility for improved therapeutic efficacy. J. Pharm. Pharmacol..

[9] Blagden N., De Matas M., Gavan P.T., York P. (2007). Crystal engineering of active pharmaceutical ingredients to improve solubility and dissolution rates. Adv. Drug Deliv. Rev..

[10] Ambrus R., Alshweiat A., Szabó-Révész P., Bartos C., Csóka I. (2022). Smartcrystals for Efficient Dissolution of Poorly Water-Soluble Meloxicam. Pharmaceutics.

[11] Csicsák D., Szolláth R., Kádár S., Ambrus R., Bartos C., Balogh E., Antal I., Köteles I., Tőzsér P., Bárdos V. (2023). The Effect of the Particle Size Reduction on the Biorelevant Solubility and Dissolution of Poorly Soluble Drugs with Different Acid-Base Character. Pharmaceutics.

[12] Jinno J.-I., Kamada N., Miyake M., Yamada K., Mukai T., Odomi M., Toguchi H., Liversidge G.G., Higaki K., Kimura T. (2006). Effect of particle size reduction on dissolution and oral absorption of a poorly water-soluble drug, cilostazol, in beagle dogs. J. Control. Release.

[13] Hermenean A., Dossi E., Hamilton A., Trotta M.C., Russo M., Lepre C.C., Sajtos C., Rusznyák Á., Váradi J., Bácskay I. (2024). Chrysin Directing an Enhanced Solubility through the Formation of a Supramolecular Cyclodextrin–Calixarene Drug Delivery System: A Potential Strategy in Antifibrotic Diabetes Therapeutics. Pharmaceuticals.

[14] Jain N.K., Gupta U. (2008). Application of dendrimer–drug complexation in the enhancement of drug solubility and bioavailability. Expert Opin. Drug Metab. Toxicol..

[15] Mu H., Holm R., Müllertz A. (2013). Lipid-based formulations for oral administration of poorly water-soluble drugs. Int. J. Pharm..

[16] Butreddy A., Bandari S., Repka M.A. (2021). Quality-by-design in hot melt extrusion based amorphous solid dispersions: An industrial perspective on product development. Eur. J. Pharm. Sci..

[17] Sripetthong S., Nalinbenjapun S., Basit A., Ovatlarnporn C. (2024). Synthesis of Quarternized Chitosans and Their Potential Applications in the Solubility Enhancement of Indomethacin by Solid Dispersion. AAPS PharmSciTech.

[18] Serajuddin A.T. (1999). Solid dispersion of poorly water-soluble drugs: Early promises, subsequent problems, and recent breakthroughs. J. Pharm. Sci..

[19] Lam M., Asare-Addo K., Nokhodchi A. (2021). Liqui-Tablet: The Innovative Oral Dosage Form Using the Newly Developed Liqui-Mass Technology. AAPS PharmSciTech.

[20] Lam M., Nokhodchi A. (2021). Producing High-Dose Liqui-Tablet (Ketoprofen 100 mg) for Enhanced Drug Release Using Novel Liqui-Mass Technology. J. Pharm. Innov..

[21] Cahyani A.N., Susanto A., Dewi I.R., Nurhikmah I. (2023). Formulasi Tablet Parasetamol Dengan Kombinasi PVP dan Amilum Umbi Porang (*Amorphopallus onchopyllus*) Sebagai Bahan Pengikat Terhadap Sifat Fisik Tablet. J. Ilm. JOPHUS J. Pharm. UMUS.

[22] Lam M., Nokhodchi A. (2019). Pharmaceutical Methods and Compositions. International Patent Application.

[23] Hillstrom C., Jakobsson J.G. (2013). Lornoxicam: Pharmacology and usefulness to treat acute postoperative and musculoskeletal pain a narrative review. Expert Opin. Pharmacother..

[24] Balfour J.A., Fitton A., Barradell L.B. (1996). Lornoxicam: A review of its pharmacology and therapeutic potential in the management of painful and inflammatory conditions. Drugs.

[25] Nousheen L., Rajasekaran S., Qureshi M.S. (2022). Solubility enhancement of lornoxicam with poloxamer 188 by solvent evaporation method. Int. J. Health Sci..

[26] Li F., Song S., Guo Y., Zhao Q., Zhang X., Pan W., Yang X. (2013). Preparation and pharmacokinetics evaluation of oral self-emulsifying system for poorly water-soluble drug Lornoxicam. Drug Deliv..

[27] Kalyanappa S., Krishna M.R., Goli D. (2015). Design and in vitro Evaluation of a Novel Sustained Release Double Layered Tablets of Lornoxicam by using semi synthetic polymers. Indian J. Pharm. Educ. Res..

[28] Nijhawan M., Santhosh A., Babu P.R.S., Subrahmanyam C.V.S. (2013). Solid state manipulation of lornoxicam for cocrystals—Physicochemical characterization. Drug Dev. Ind. Pharm..

[29] Zewail M.B., ASaad G.F., Swellam S.M., Abd-Allah S.M., K.HOsny S., Sallah S.K., E.EIssa J., S.MOhamed S., El-Dakroury W.A. (2022). Design, characterization and in vivo performance of solid lipid nanoparticles (SLNs)-loaded mucoadhesive buccal tablets for efficient delivery of Lornoxicam in experimental inflammation. Int. J. Pharm..

[30] Usman F., Javed I., Hussain S.Z., Ranjha N.M., Hussain I. (2016). Hydrophilic nanoparticles packed in oral tablets can improve the plasma profile of short half-life hydrophobic drugs. RSC Adv..

[31] Shokri J., Adibkia K., Javadzadeh Y. (2015). Liquisolid Technology: What It Can Do for NSAIDs Delivery?. Colloids Surf. B Biointerfaces.

[32] El-Setouhy D.A., Gamiel A.A.-R., Badawi A.A.E.-L., Osman A.S., Labib D.A. (2016). Comparative study on thein vitroperformance of blister molded and conventional lornoxicam immediate release liquitablets: Accelerated stability study and anti-inflammatory and ulcerogenic effects. Pharm. Dev. Technol..

[33] Strickley R.G. (2004). Solubilizing Excipients in Oral and Injectable Formulations. Pharm. Res..

[34] Hussein A.A. (2017). Preparation and Evaluation of Liquid and Solid Self-Microemulsifying Drug Delivery System of Mebendazole. Iraqi J. Pharm. Sci..

[35] Kolling W.M. (2004). Handbook of Pharmaceutical Excipients. Am. J. Pharm. Educ..

[36] Wang Y., Chin C.-Y., Shivashekaregowda N.K.H., Shi Q. (2024). Effects of polyvinylpyrrolidone on the crystallization of amorphous griseofulvin: Fracture and molecular mobility. J. Appl. Crystallogr..

[37] Kumar R., Sinha V. (2011). Tailoring of drug delivery of 5-fluorouracil to the colon via a mixed film coated unit system. Acta Pharm..

[38] Humbert-Droz P., Seidel M., Martani R. (2000). Fast Disintegrating Oral Dosage Form. U.S. Patent.

[39] Carstensen J.T. (2002). Preformulation. Modern Pharmaceutics.

[40] Wagner J.G. (1969). Interpretation of Percent Dissolved-Time Plots Derived from In Vitro Testing of Conventional Tablets and Capsules. J. Pharm. Sci..

[41] Moore J.W., Flanner H.H. (1996). Mathematical Comparison of Dissolution Profiles. Pharm. Technol..

[42] Moutasim M.Y., ElMeshad A.N., El-Nabarawi M.A. (2017). A pharmaceutical study on lornoxicam fast disintegrating tablets: Formulation and in vitro and in vivo evaluation. Drug Deliv. Transl. Res..

[43] Sheinin E.B. (2025). Pharmacopeial Methods and Tests. Specification of Drug Substances and Products.

[44] Wesolowski M., Leyk E. (2023). Coupled and Simultaneous Thermal Analysis Techniques in the Study of Pharmaceuticals. Pharmaceutics.

[45] He Y., Majid K., Maqbool M., Hussain T., Yousaf A.M., Khan I.U., Mehmood Y., Aleem A., Arshad M.S., Younus A. (2020). Formulation and characterization of lornoxicam-loaded cellulosic-microsponge gel for possible applications in arthritis. Saudi Pharm. J..

[46] Carvalho A., Zangaro G., Fernandes R., Ekawa B., Nascimento A., Silva B., Ashton G., Parkes G., Ionashiro M., Caires F. (2019). Lornoxicam drug—A new study of thermal degradation under oxidative and pyrolysis conditions using the thermoanalytical techniques, DRX and LC-MS/MS. Thermochim. Acta.

[47] Suresh K., Nangia A. (2014). Lornoxicam Salts: Crystal Structures, Conformations, and Solubility. Cryst. Growth Des..

[48] Khairnar S., Kini R., Harwalkar M., Chaudhari S.R. (2014). A Review on Freeze Drying Process of Pharmaceuticals. Int. J. Res. Pharm. Sci..

[49] Kumbhar D., Wavikar P., Vavia P. (2013). Niosomal Gel of Lornoxicam for Topical Delivery: In vitro Assessment and Pharmacodynamic Activity. AAPS PharmSciTech.

[50] Colombo M., Orthmann S., Bellini M., Staufenbiel S., Bodmeier R. (2017). Influence of Drug Brittleness, Nanomilling Time, and Freeze-Drying on the Crystallinity of Poorly Water-Soluble Drugs and Its Implications for Solubility Enhancement. AAPS PharmSciTech.

[51] Kurakula M., Rao G.K. (2020). Pharmaceutical assessment of polyvinylpyrrolidone (PVP): As excipient from conventional to controlled delivery systems with a spotlight on COVID-19 inhibition. J. Drug Deliv. Sci. Technol..

[52] Rusdin A., Gazzali A.M., Thomas N.A., Megantara S., Aulifa D.L., Budiman A., Muchtaridi M. (2024). Advancing Drug Delivery Paradigms: Polyvinyl Pyrolidone (PVP)-Based Amorphous Solid Dispersion for Enhanced Physicochemical Properties and Therapeutic Efficacy. Polymers.

[53] Ochi M., Kimura K., Kanda A., Kawachi T., Matsuda A., Yuminoki K., Hashimoto N. (2015). Physicochemical and Pharmacokinetic Characterization of Amorphous Solid Dispersion of Meloxicam with Enhanced Dissolution Property and Storage Stability. AAPS PharmSciTech.

[54] Srivastava A., Khan M.A., Bedi S., Bhandari U. (2022). A Review on Different Solubility Enhancement Techniques of Ticagrelor. Int. J. Pharm. Investig..

[55] Jadhav P., Yadav A. (2019). Formulation, optimization, and in vitro evaluation of polymeric nanosuspension of flurbiprofen. Asian J. Pharm. Clin. Res..

[56] Joseph J., Vedha Hari B.N., Devi D.R. (2018). Experimental optimization of Lornoxicam liposomes for sustained topical delivery. Eur. J. Pharm. Sci..

[57] Noreen M., Farooq M.A., Ghayas S., Bushra R., Yaqoob N., Abrar M.A. (2019). Formulation and in vitro characterization of sustained release tablets of lornoxicam. Lat. Am. J. Pharm..

[58] Alhalaweh A., Vilinska A., Gavini E., Rassu G., Velaga S.P. (2011). Surface Thermodynamics of Mucoadhesive Dry Powder Formulation of Zolmitriptan. AAPS PharmSciTech.

[59] Liu T., Hao J., Yang B., Hu B., Cui Z., Li S. (2018). Contact Angle Measurements: An Alternative Approach Towards Understanding the Mechanism of Increased Drug Dissolution from Ethylcellulose Tablets Containing Surfactant and Exploring the Relationship Between Their Contact Angles and Dissolution Behaviors. AAPS PharmSciTech.

[60] Hanum T.I., Nasution A., Sumaiyah S., Bangun H. (2023). Physical stability and dissolution of ketoprofen nanosuspension formulation: Polyvinylpyrrolidone and Tween 80 as stabilizers. Pharmacia.

[61] Bansal A.K., Pande V. (2013). Development and Evaluation of Dual Cross-Linked Pulsatile Beads for Chronotherapy of Rheumatoid Arthritis. J. Pharm..

[62] Taldaev A., Pankov D.I., Terekhov R.P., Zhevlakova A.K., Selivanova I.A. (2023). Modification of the Physicochemical Properties of Active Pharmaceutical Ingredients via Lyophilization. Pharmaceutics.

[63] Patil S.B., Shahi S.R., Udavant Y.K., Atram S.C., Salunke R.J., Neb G.B. (2009). Formulation and evaluation of quick dispersible tablet of olanzapine. Int. J. Pharm. Res. Dev.-Online.

[64] Rajebahadur M., Zia H., Nues A., Lee C. (2006). Mechanistic Study of Solubility Enhancement of Nifedipine Using Vitamin E TPGS or Solutol HS-15. Drug Deliv..

